# Post-Marketing Safety Profile of Mirikizumab: A Multi-Database Pharmacovigilance Study Using FAERS and JADER with IL-23 Inhibitor Class Comparison

**DOI:** 10.3390/bioengineering13070789

**Published:** 2026-07-09

**Authors:** Jeong-Gyu Choi, Eun Jeong Gong, Chang Seok Bang, Jae Jun Lee

**Affiliations:** 1 Institute of New Frontier Research, Hallym University College of Medicine, Chuncheon 24253, Republic of Koreagong-eun@hanmail.net (E.J.G.); iloveu59@hallym.or.kr (J.J.L.); 2 Department of Internal Medicine, Hallym University College of Medicine, Chuncheon 24253, Republic of Korea; 3 Department of Anesthesiology and Pain Medicine, Hallym University College of Medicine, Chuncheon 24253, Republic of Korea

**Keywords:** mirikizumab, disproportionality analysis, hepatotoxicity, IL-23 inhibitor, ulcerative colitis, Crohn’s disease

## Abstract

Background: Mirikizumab, a first-in-class interleukin-23p19 antagonist, was approved for ulcerative colitis (2023) and Crohn’s disease (2025). The US Food and Drug Administration (FDA) identified a hepatotoxicity signal during pre-approval review, mandating post-marketing surveillance. No independent pharmacovigilance analysis has been published. Aims: To characterise the post-marketing safety profile of mirikizumab using multi-database pharmacovigilance, with a focus on hepatotoxicity and IL-23 inhibitor class comparison. Methods: Disproportionality analysis of the FDA Adverse Event Reporting System (FAERS; Q4 2023–Q4 2025) and Japanese Adverse Drug Event Report database (JADER) was performed using four algorithms (reporting odds ratio, proportional reporting ratio, Bayesian confidence propagation neural network, empirical Bayesian geometric mean). Signals of disproportionate reporting were defined by concordance of all four methods. Active comparator analysis against risankizumab, guselkumab and ustekinumab, Weibull time-to-onset modelling and hepatotoxicity case characterisation were conducted. Reporting followed READUS-PV guidelines. Results: We identified 564 mirikizumab reports in FAERS and 123 in JADER. Nine signals met all four criteria in FAERS, including spontaneous abortion (Reporting odds ratio (ROR) 10.16, 95% CI 5.16–20.02), pulmonary embolism (ROR 5.56, 2.93–10.56) and injection site reactions. Hepatotoxicity showed no disproportionate reporting in either FAERS (ROR 1.19, 0.74–1.92; *n* = 17) or JADER (ROR 0.24, 0.05–1.19; *n* = 1). Comparator analysis identified cytomegalovirus infection and interstitial lung disease as mirikizumab-specific versus the IL-23 class. Weibull analysis (β = 0.65) indicated early-onset adverse event clustering. Discussion: This first multi-database pharmacovigilance study of mirikizumab did not confirm the FDA-flagged hepatotoxicity signal. Potential signals warranting further investigation include thromboembolic events and pulmonary toxicity.

## 1. Introduction

Interleukin-23 (IL-23) is a heterodimeric cytokine comprising p19 and p40 subunits that plays a central role in the pathogenesis of inflammatory bowel disease (IBD) through activation of the IL-23/IL-17 immune axis [1,2]. Genome-wide association studies identified IL23R as a major IBD susceptibility gene [3], establishing the biological rationale for therapeutic targeting of this pathway. The IL-23 inhibitor class has expanded rapidly, with ustekinumab (anti-IL-12/23p40), risankizumab and guselkumab (anti-IL-23p19) demonstrating efficacy across IBD phenotypes [4,5].

Mirikizumab (Omvoh^®^; Eli Lilly, Indianapolis, IN, USA) is a humanised IgG4 monoclonal antibody that selectively targets the p19 subunit of IL-23. It received its first global approval in Japan for ulcerative colitis (UC) in March 2023, followed by US FDA approval in October 2023 based on the LUCENT-1 and LUCENT-2 phase 3 trials [6,7]. Subsequently, FDA approval for Crohn’s disease (CD) was granted in January 2025 based on the VIVID-1 trial [8]. The 2024 American Gastroenterology Association (AGA) living guideline on UC management conditionally recommends mirikizumab alongside other advanced therapies [9].

During pre-approval review, the FDA’s Division of Hepatology and Nutrition Drug-Induced Liver Injury (DILI) team identified a hepatotoxicity signal after evaluating 34 subjects with liver biochemistry elevations across the clinical development programme [10]. Five cases were assessed as at least “possible mirikizumab DILI,” including one Hy’s Law case with alanine aminotransferase (ALT) 18× the upper limit of normal (ULN) and total bilirubin 2.4× ULN [10]. The DILI team concluded that mirikizumab “carries a hepatotoxicity risk that is likely immune-mediated.” Consequently, the FDA mandated post-marketing requirement (PMR) 4409-4, an observational study to assess severe acute liver injury, and required enhanced pharmacovigilance with 15-day alert reporting for all hepatotoxicity cases for three years post-approval [10,11].

Spontaneous reporting system-based pharmacovigilance studies have been published for other IL-23 class agents, including risankizumab [12,13,14], guselkumab [15], and ustekinumab [16,17,18], providing class-level safety context. However, no pharmacovigilance analysis of mirikizumab from any spontaneous reporting database has been published to date. Given the FDA’s hepatotoxicity concern and the expanding clinical use of mirikizumab across IBD indications, an independent post-marketing safety assessment is warranted.

This study aimed to characterise the post-marketing safety profile of mirikizumab through disproportionality analysis of the FDA Adverse Event Reporting System (FAERS) and the Japanese Adverse Drug Event Report database (JADER), with particular focus on hepatotoxicity signal evaluation, active comparator reference disproportionality analysis (ACR-DA) against the IL-23/IL-12/23 inhibitor class, and Weibull time-to-onset modelling.

## 2. Materials and Methods

### 2.1. Data Sources

This study analysed two spontaneous reporting databases. The FAERS database, maintained by the US FDA, is the largest global pharmacovigilance database and contains adverse event reports submitted by healthcare professionals, consumers, and manufacturers [19]. We extracted FAERS quarterly data files from Q4 2023 (the quarter of mirikizumab’s FDA approval) through Q4 2025, comprising 2,875,136 deduplicated primary suspect reports. JADER, maintained by Japan’s Pharmaceuticals and Medical Devices Agency (PMDA), was analysed using data available through January 2026, containing approximately 1,008,612 reports [20,21]. Canada Vigilance was queried but yielded zero mirikizumab reports and was excluded from further analysis. This study used publicly available, de-identified data and did not require ethics committee approval.

### 2.2. Drug Identification and Case Selection

Mirikizumab cases were identified using generic name (“mirikizumab”) and brand name (“Omvoh”) search terms in drug files, restricted to primary suspect (PS) designation. Comparator drugs included risankizumab (Skyrizi^®^, AbbVie Inc., North Chicago, IL, USA), guselkumab (Tremfya^®^, Janssen Biotech, Inc., Horsham, PA, USA), and ustekinumab (Stelara^®^, Janssen Biotech, Inc., Horsham, PA, USA). Case deduplication was performed using the CASEID/primaryid methodology, retaining the most recent report version for each unique case [19].

### 2.3. Disproportionality Analysis

Four complementary signal detection algorithms were applied:

(i) Reporting odds ratio (ROR): signal threshold defined as lower 95% confidence interval (CI) > 1 and case count ≥ 3 [22]; (ii) Proportional reporting ratio (PRR): signal defined as PRR ≥ 2, χ^2^ ≥ 4, and case count ≥ 3 [23]; (iii) Bayesian confidence propagation neural network (BCPNN): signal defined as information component lower 95% credible interval (IC025) > 0 [24]; (iv) empirical Bayesian geometric mean (EBGM) from the multi-item gamma Poisson shrinker: signal defined as lower 90% one-sided CI (EB05) > 2 [25]. A signal of disproportionate reporting (SDR) was defined as concordance of all four algorithms, maximising specificity [26].

### 2.4. Primary and Comparator Analyses

The primary analysis compared mirikizumab reports against the full FAERS background (all other drugs). The Active-comparator restricted disproportionality analysis (ACR-DA) compared mirikizumab against the combined IL-23/IL-12/23 inhibitor class (risankizumab + guselkumab + ustekinumab) to identify mirikizumab-specific signals beyond class effects. JADER analysis was conducted independently using the same four-algorithm approach.

### 2.5. Hepatotoxicity Analysis

Hepatotoxicity was assessed using a composite endpoint comprising Standardised MedDRA Query (SMQ) preferred terms (PTs) for hepatobiliary disorders [27], including DILI, hepatotoxicity, hepatic failure, hepatic enzyme increased, ALT increased, aspartate aminotransferase (AST) increased, cholestasis, jaundice, and liver disorder. This composite analysis was performed across all four drugs in both FAERS and JADER. Individual case review was conducted for all mirikizumab-associated hepatotoxicity reports, extracting demographics, clinical presentation, concomitant medications, and outcomes.

### 2.6. Weibull Time-to-Onset Analysis

Time-to-onset (TTO) was calculated as the interval between the event date and the FDA report date for cases with valid date information. The Weibull distribution was fitted to TTO data using maximum likelihood estimation. The shape parameter (β) was interpreted as follows: β < 1 indicates an early failure (decreasing hazard) pattern suggesting adverse events cluster early after treatment initiation; β = 1 indicates a random (constant hazard) pattern; β > 1 indicates a wear-out (increasing hazard) pattern [28,29].

### 2.7. Reporting Guidelines

This study was reported in accordance with the READUS-PV (REporting of A Disproportionality Analysis for DrUg Safety Signal Detection Using Individual Case Safety Reports in PharmacoVigilance) guidelines [30,31].

### 2.8. Sensitivity Analyses

Four prespecified sensitivity analyses were conducted to assess the robustness of the primary findings. First, an indication-restricted analysis repeated the primary disproportionality analysis among mirikizumab reports with UC as the recorded indication, thereby minimising confounding by indication heterogeneity. Second, age-stratified analyses were performed within subgroups defined as ≤64 years and >64 years for a prespecified set of clinically relevant preferred terms (ulcerative colitis, injection site pain, pulmonary embolism, spontaneous abortion, maternal exposure during pregnancy, frequent bowel movements, underdose, cytomegalovirus infection, and interstitial lung disease). Third, sex-stratified analyses were performed for the same preferred terms, excluding female-specific events for the male subgroup. Fourth, quarterly reporting trends for mirikizumab were examined to evaluate the potential contribution of the Weber effect (stimulated reporting in the first two years following approval).

A CD-restricted analysis was not undertaken because the CD indication was approved only in January 2025, providing less than one year of post-marketing exposure within the study period and an insufficient number of CD-coded reports for reliable disproportionality estimation.

## 3. Results

### 3.1. Case Identification and Demographics

We identified 564 mirikizumab reports as primary suspects in FAERS (Q4 2023–Q4 2025) and 123 in JADER. Comparator case counts in FAERS were 35,660 for risankizumab, 11,268 for guselkumab, and 10,943 for ustekinumab. Table 1 summarises the demographic characteristics. Among mirikizumab reports, 42.9% were female, with a median age of 49 years (interquartile range [IQR] 34–65). The US accounted for 55.5% (*n* = 313) of reports. The mirikizumab cohort was younger than the risankizumab cohort (median 49 vs. 61 years), consistent with the UC/CD indication versus the predominantly dermatological indications of risankizumab. The sex distribution of mirikizumab was comparable to that of guselkumab (42.3% female) and ustekinumab (41.7% female), while risankizumab showed a higher female proportion (53.2%).

### 3.2. Primary Disproportionality Analysis: FAERS

Among 409 PTs reported for mirikizumab, nine met the SDR criteria with concordance of all four algorithms (Table 2; complete list of all 28 ROR-positive signals in Table S1). The strongest signal was for ulcerative colitis (*n* = 78; ROR 38.41, 95% CI 30.24–48.78), representing the underlying disease indication. Injection site reactions comprised three SDRs: injection site pain (*n* = 62; ROR 6.27), injection site discomfort (*n* = 5; ROR 13.21), and injection site hypersensitivity (*n* = 3; ROR 22.38), consistent with the known safety profile from clinical trials where injection site reactions occurred in 9–10% of mirikizumab-treated patients [6,8].

**Figure 1:**
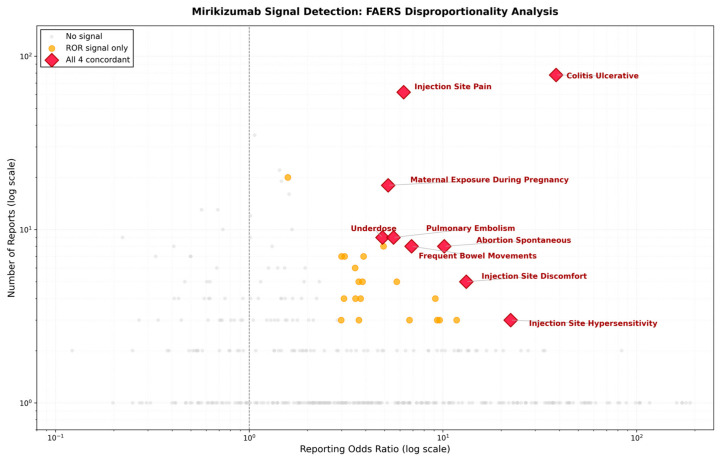
Signal detection landscape for mirikizumab in FAERS.

Two novel signals merit particular attention. Spontaneous abortion (*n* = 8; ROR 10.16, 95% CI 5.16–20.02) was identified alongside maternal exposure during pregnancy (*n* = 18; ROR 5.22, 95% CI 3.28–8.29), consistent with the contraindicated use of mirikizumab during pregnancy per the prescribing information [11]. Pulmonary embolism (*n* = 9; ROR 5.56, 95% CI 2.93–10.56) represented a potentially novel thromboembolic signal not previously described for IL-23 inhibitors. Underdose (*n* = 9; ROR 4.88) and frequent bowel movements (*n* = 8; ROR 6.89) were also identified as SDRs but are clinically attributable to medication error and underlying IBD, respectively. Figure 1 presents the signal landscape as a bubble plot showing the relationship between ROR magnitude and report count.

Bubble plot showing the relationship between reporting odds ratio (x-axis, log scale) and number of reports (y-axis, log scale) for all preferred terms. Red diamonds indicate signals meeting all four algorithm criteria (ROR, PRR, BCPNN, EBGM concordant); orange circles indicate ROR-only signals; grey circles indicate no signal. The dashed vertical line represents ROR = 1 (null). Nine signals met the all-four-concordance criterion. ROR, reporting odds ratio; BCPNN, Bayesian confidence propagation neural network; EBGM, empirical Bayesian geometric mean.

### 3.3. Active Comparator Analysis: Mirikizumab vs. IL-23 Class

When mirikizumab was compared against the combined IL-23/IL-12/23 inhibitor class, 12 PTs met the SDR criteria (Table 3; complete ACR-DA signal list in Table S2). The ACR-DA confirmed the pulmonary embolism signal (*n* = 9; ROR 5.96, 95% CI 3.08–11.53) as disproportionately associated with mirikizumab relative to the class. Two pulmonary signals were identified: pulmonary toxicity (*n* = 3; ROR 240.48) and interstitial lung disease (ILD; *n* = 5; ROR 12.49, 95% CI 5.14–30.38). Cytomegalovirus (CMV) infection (*n* = 4; ROR 26.54, 95% CI 9.39–75.04) emerged as a mirikizumab-specific signal relative to the class, a finding of clinical relevance given the association between CMV reactivation and IBD flares [32]. Injection site reactions showed higher disproportionality for mirikizumab than the class average. Medication error-related signals (accidental underdose, incorrect dose administered) likely reflect the early post-marketing phase with a novel dosing regimen (intravenous induction followed by subcutaneous maintenance).

### 3.4. Results of Hepatotoxicity Analaysis

**Figure 2:**
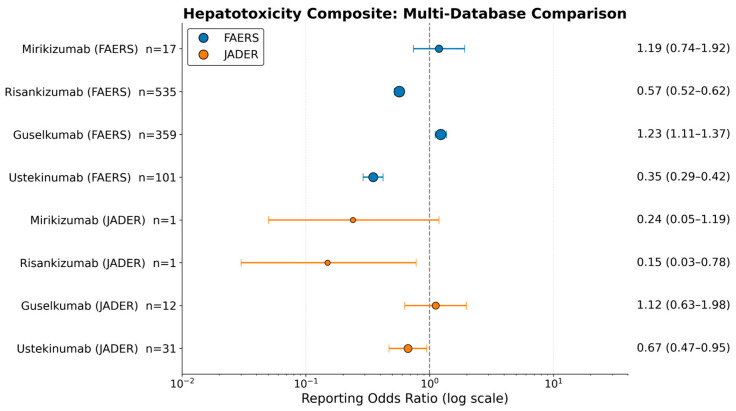
Hepatotoxicity composite analysis: multi-database forest plot.

The composite hepatotoxicity analysis revealed no SDR for mirikizumab in either database (Table 4, Figure 2). In FAERS, 17 hepatotoxicity reports were identified (ROR 1.19, 95% CI 0.74–1.92), and in JADER, only one report was identified (ROR 0.24, 95% CI 0.05–1.19). None of the four algorithms met their respective signal thresholds in either database. Among the comparator drugs, risankizumab (*n* = 535; ROR 0.57) and ustekinumab (*n* = 101; ROR 0.35) showed reduced reporting relative to background, while guselkumab (*n* = 359; ROR 1.23, 95% CI 1.11–1.37) showed a modestly elevated ROR with the lower CI crossing 1 but did not meet all four algorithm criteria. Individual hepatic preferred term results across all four drugs are provided in Table S3.

Reporting odds ratios with 95% confidence intervals for the hepatotoxicity composite endpoint across mirikizumab, risankizumab, guselkumab, and ustekinumab in both FAERS (blue) and JADER (orange). The dashed vertical line represents ROR = 1 (null). Circle size is proportional to case count. No drug met the signal threshold in either database using all four algorithms. ROR, reporting odds ratio; FAERS, FDA Adverse Event Reporting System; JADER, Japanese Adverse Drug Event Report database.

A case-level review of the 17 mirikizumab hepatotoxicity reports revealed the following: countries of origin were the US (*n* = 10), Japan (*n* = 3), Germany (*n* = 2), Switzerland (*n* = 1), and Austria (*n* = 1). Sex distribution was female (*n* = 8), male (*n* = 5), and unknown (*n* = 4), with a median age of approximately 55 years (range 38–79). The most frequently reported hepatic PTs included AST increased (*n* = 3), hepatic enzyme increased (*n* = 3), drug-induced liver injury (*n* = 2), ALT increased (*n* = 2), cholestasis (*n* = 2), and liver disorder (*n* = 2). UC was the stated indication in 10 cases, CD in one, and IBD (unspecified) in one; five cases had unknown indications. One case of autoimmune hepatitis was reported in a 38-year-old male from Germany, and one case of hepatic failure was reported from the US. Several cases involved concomitant hepatotoxic medications, including budesonide, azathioprine, mesalamine, and prednisolone, complicating causal attribution. Full case-level details are provided in Table S4.

### 3.5. JADER Analysis

Among 123 mirikizumab reports in JADER, two PTs met the SDR criteria: ulcerative colitis (*n* = 31; ROR 250.29, 95% CI 166.47–376.32) and drug intolerance (*n* = 4; ROR 135.07, 95% CI 52.32–348.71). The very high ROR for UC in JADER, compared with FAERS (ROR 38.41), likely reflects the smaller denominator and the fact that mirikizumab was approved earlier in Japan, resulting in a higher proportion of UC-related reports. Hepatotoxicity showed no signal in JADER (*n* = 1; ROR 0.24, 95% CI 0.05–1.19), concordant with the FAERS finding. The complete JADER signal list is provided in Table S5. The drug intolerance signal observed exclusively in JADER and not replicated in FAERS may reflect differences in reporting culture, ethnicity-related variability in immunogenicity or pharmacokinetics, and differences in dosing or monitoring practices between the two regions.

### 3.6. Time-to-Onset Analysis

**Figure 3:**
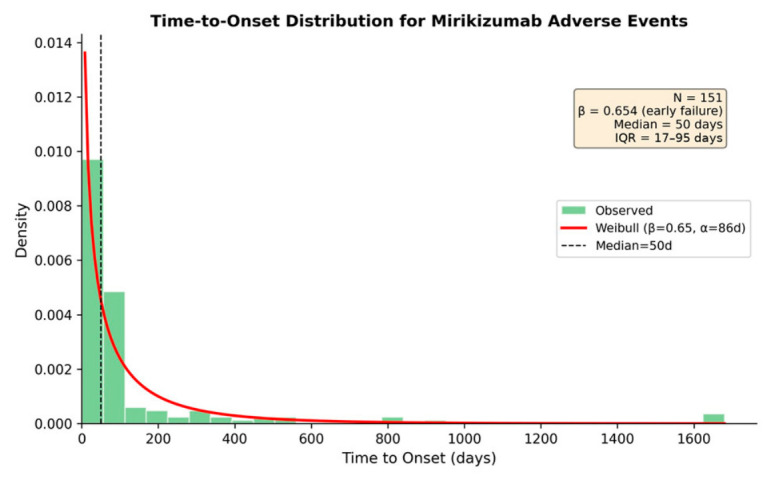
Time-to-onset distribution for mirikizumab adverse events.

Valid TTO data were available for 151 of 564 reports (26.8%). The median TTO was 50 days (IQR 17–95 days). Weibull distribution fitting yielded a shape parameter β = 0.654 (scale parameter α = 86.2 days), indicating an early failure pattern (Figure 3). This β < 1 value signifies that the hazard of adverse event reporting is highest immediately after treatment initiation and decreases over time, consistent with early immune-mediated or infusion/injection-related reactions dominating the safety profile during the induction phase. This pattern aligns with the mirikizumab dosing regimen, which involves higher-dose intravenous infusions during the 12-week induction period followed by lower-dose subcutaneous maintenance injections.

Histogram of observed time-to-onset values (green bars) with fitted Weibull distribution curve (red line). N = 151 reports with valid time-to-onset data. The Weibull shape parameter β = 0.654 indicates an early failure pattern; the scale parameter α = 86.2 days. Median time-to-onset = 50 days (IQR 17–95 days). The dashed vertical line indicates the median. IQR, interquartile range.

### 3.7. Results of Sensitivity Analyses

The indication-restricted analysis, confined to mirikizumab reports with UC as the recorded indication, yielded 12 signals meeting the all-four-algorithm criterion (Table S6). All nine signals identified in the primary analysis were reproduced, confirming the robustness of the main findings. Three additional signals emerged in the UC-restricted analysis: pulmonary toxicity (*n* = 3; ROR 14.72, 95% CI 4.72–45.89), CMV infection (*n* = 4; ROR 12.24, 4.57–32.82) and deep vein thrombosis (*n* = 5; ROR 7.90, 3.27–19.10), strengthening the thromboembolic and pulmonary signals observed in the primary and active comparator analyses.

Age-stratified analyses (Table S7) revealed that the pulmonary embolism signal was driven predominantly by older patients (>64 years: *n* = 4; ROR 7.97, 95% CI 2.94–21.64, all-four-concordant), whereas in patients ≤64 years the signal did not meet statistical significance (*n* = 3; ROR 3.05, 0.98–9.52). Conversely, the injection site pain signal was concentrated in younger patients (≤64 years: *n* = 38; ROR 4.96, 3.52–6.98, all-four-concordant) and was not detected in the older subgroup. The spontaneous abortion signal remained robust in the ≤64-year subgroup (*n* = 6; ROR 9.36, 4.17–21.03, all-four-concordant).

Sex-stratified analyses (Table S8) demonstrated that the pulmonary embolism signal was present in both sexes (female: *n* = 4; ROR 5.61, 95% CI 2.09–15.08; male: *n* = 4; ROR 4.66, 1.73–12.54), with both meeting the all-four-algorithm criterion. The injection site pain signal was stronger in females (*n* = 40; ROR 7.60, 5.42–10.67) than in males (*n* = 13; ROR 3.30, 1.88–5.78), although both met signal criteria. The CMV infection signal reached all-four-concordance in males only (*n* = 3; ROR 11.44, 3.66–35.80). As expected, spontaneous abortion and maternal exposure during pregnancy signals were confined to the female subgroup.

Quarterly reporting trend analysis (Figure S1) showed that mirikizumab report volume increased from 21 reports in Q4 2023 to a peak of 134 reports in Q3 2024, followed by relative stabilisation at 84–119 reports per quarter through Q4 2025. The delayed peak (approximately 9–12 months after the first reporting quarter) rather than an immediate post-approval surge suggests that the Weber effect (stimulated reporting shortly after launch) had limited influence on the observed signals and that reporting patterns reflect progressive clinical uptake and the expanded CD indication approved in 2025. Quarterly counts in this analysis were derived from drug-record matches across quarterly files; the apparent total exceeds the deduplicated primary-suspect cohort because individual cases reported across multiple quarters (e.g., follow-up reports) are counted in each quarter of submission.

## 4. Discussion

This study represents the first pharmacovigilance analysis of mirikizumab from any spontaneous reporting database, providing real-world safety data to complement clinical trial evidence. Our principal finding is that the FDA-flagged hepatotoxicity signal was not confirmed through disproportionality analysis of either FAERS or JADER, a result that is clinically reassuring for prescribers managing patients with IBD.

The absence of a hepatotoxicity SDR in FAERS (ROR 1.19, 95% CI 0.74–1.92) despite 17 reports contrasts with the DILI team’s pre-approval concern [10]. Several factors may account for this discrepancy. First, the DILI assessment was based on a controlled clinical trial population with systematic liver biochemistry monitoring, whereas FAERS captures spontaneously reported events subject to underreporting [19]. Second, the comparatively short post-marketing exposure period (approximately two years) may be insufficient to generate a detectable signal for a potentially rare event. Third, the enhanced pharmacovigilance requirement—mandating 15-day alert reporting for hepatotoxicity cases—may paradoxically distribute hepatic events across specific PTs rather than concentrating them, potentially diluting any composite signal. This finding does not exclude hepatotoxicity as a genuine risk; the PMR 4409-4 study, with a final report expected by 2037, will provide definitive epidemiological data [10,11].

The cross-database concordance between FAERS and JADER for the negative hepatotoxicity finding strengthens the interpretation. Both databases independently showed ROR point estimates compatible with no disproportionate reporting. Among comparator drugs, guselkumab showed the highest hepatotoxicity ROR (1.23, 95% CI 1.11–1.37 in FAERS), although this did not meet all four algorithm criteria. This differential within the IL-23 class warrants monitoring but may reflect confounding by indication, as guselkumab is used across psoriasis, psoriatic arthritis, and UC, with varying baseline hepatotoxicity risks [15,33].

The identification of spontaneous abortion as an SDR (ROR 10.16) should be interpreted in context. Mirikizumab, like all anti-IL-23 agents, is contraindicated during pregnancy, and the signal likely reflects reporting of known pregnancy exposures rather than an unexpected pharmacological effect [11]. The PIANO registry has demonstrated that biologic therapies as a class are not associated with increased rates of adverse pregnancy outcomes in IBD [34,35]; however, mirikizumab-specific pregnancy data remain limited.

The pulmonary embolism signal (ROR 5.56 in primary analysis; ROR 5.96 in ACR-DA) has not previously been reported. Thromboembolic events have not been a recognised class effect of IL-23 inhibitors, although a cerebrovascular accident signal was reported for risankizumab in a prior FAERS analysis [13]. The biological plausibility of IL-23 blockade promoting thrombosis is uncertain, and confounding by the prothrombotic state associated with active IBD represents the most likely alternative explanation [36]. Nevertheless, this signal warrants continued surveillance.

The ACR-DA identified two notable mirikizumab-specific pulmonary signals—pulmonary toxicity (ROR 240.48) and ILD (ROR 12.49)—neither of which has been reported for other IL-23 inhibitors. A meta-analysis of 54 randomised controlled trials found no increased ILD risk with IL-12/23 or IL-23 antagonists [37], suggesting these signals may reflect reporting artefacts or confounders rather than true pharmacological effects. The very wide confidence intervals and small case counts (*n* = 3 and *n* = 5, respectively) further limit interpretation. CMV infection (*n* = 4; ROR 26.54 vs. class) is clinically noteworthy given the established relationship between CMV reactivation and IBD flares, particularly in the setting of immunosuppression [32]. Whether mirikizumab confers differential CMV reactivation risk compared with other IL-23 inhibitors requires dedicated investigation. Given the very small case counts and the absence of an established biological pathway by which IL-23 blockade would be expected to alter pulmonary parenchymal or cytomegalovirus-related immune surveillance [1,2,32,37], these findings should be regarded as potential signals requiring further validation rather than confirmed adverse drug reactions.

The Weibull analysis revealing an early failure pattern (β = 0.654) has practical implications. The clustering of adverse events in the initial weeks following treatment initiation aligns with the mirikizumab induction regimen, which involves three 300 mg intravenous infusions at weeks 0, 4, and 8, followed by 200 mg subcutaneous injections from week 12 [6,8]. This pattern suggests that enhanced monitoring during induction may be prudent and supports the clinical observation that injection site reactions and early tolerability issues predominate during the initial treatment phase. The shift to subcutaneous maintenance appears to reduce the adverse event reporting rate, consistent with the concept of immune adaptation.

### 4.1. Strengths and Limitations

This study has several strengths. It is the first multi-database pharmacovigilance analysis of mirikizumab, employing four complementary signal detection algorithms with a conservative all-four-concordance SDR definition. The ACR-DA design provides drug-specific signal identification beyond class effects. The inclusion of JADER provides geographic diversity and cross-database validation. Reporting followed the READUS-PV guidelines [30,31]. The robustness of the primary findings was confirmed through four prespecified sensitivity analyses, including indication-restricted, age-stratified, sex-stratified, and temporal reporting analyses.

Limitations inherent to spontaneous reporting system-based pharmacovigilance must be acknowledged. Spontaneous reporting is subject to underreporting, reporting bias, and the Weber effect (reporting peaks in the first two years after approval) [19]. Disproportionality analysis cannot establish causality, calculate incidence rates, or control for confounders such as disease severity and concomitant medications. The relatively small number of mirikizumab reports (*n* = 564) limits statistical power for rare events. The absence of denominator data (actual patient exposure) prevents incidence estimation. The low TTO data availability (26.8%) limits the generalisability of the Weibull analysis. Finally, the two-year post-marketing window may be insufficient to detect signals for adverse events with long latency periods. The 73.2% missing rate may introduce selection bias if reports with documented event and reporting dates differ systematically from those without; the estimated shape parameter (β) should therefore be interpreted with caution, and analyses with more complete time-to-onset ascertainment—ideally after at least five years of post-marketing follow-up—will be required to confirm the early-failure pattern. Although the four-way concordance criterion considerably increases specificity, the testing of 409 preferred terms raises the possibility of residual false-positive findings; we did not formally adjust for multiple comparisons because the conservative concordance requirement and the active comparator framework partially mitigate this concern, but signals based on small case counts should be regarded as hypothesis-generating.

Several pharmaceutical and biopharmaceutical dimensions were also beyond the scope of the available spontaneous reporting data and represent further limitations. First, adverse events could not be stratified by route of administration, although mirikizumab employs intravenous induction (300 mg at weeks 0, 4 and 8) followed by subcutaneous maintenance (200 mg every 4 weeks); the two routes differ in bioavailability, peak concentration and local-reaction profile, which is particularly relevant to the pulmonary embolism, injection site and underdose signals. Second, FAERS and JADER do not capture anti-drug antibody status, so the identified signals could not be correlated with the immunogenicity of this humanised IgG4 antibody or compared with fully human agents (risankizumab, guselkumab). Third, the underdose signal could not be examined in relation to delivery-device characteristics, solution viscosity, injection volume or country-level device differences. Fourth, mirikizumab is dosed at a fixed amount without weight adjustment, yet body-weight and exposure data are not recorded in these databases, precluding analysis of weight-related exposure variability and its potential link to early, immune-mediated events; correspondingly, the Weibull early-onset pattern (β = 0.654) could not be mapped onto the induction versus maintenance pharmacokinetic phases. Fifth, formulation-dependent factors (pH, osmolality, excipients, viscosity) that may underlie injection-site reactions could not be compared across products. Finally, body-weight distribution was unavailable, and only aggregate sex data were reported (Table 1), limiting weight- and sex-stratified signal analysis beyond the sensitivity analyses presented. These aspects warrant dedicated pharmacokinetic and formulation-oriented investigation and should be addressed in future studies with access to individual exposure, device and immunogenicity data.

### 4.2. Conclusions

In conclusion, this first multi-database pharmacovigilance study of mirikizumab provides reassurance regarding the FDA-flagged hepatotoxicity concern, with no disproportionate hepatotoxicity reporting detected in either FAERS or JADER. Potential signals warranting further investigation, including pulmonary embolism, CMV infection, and ILD relative to the IL-23 class, warrant continued post-marketing surveillance. The early failure pattern identified by Weibull analysis supports enhanced monitoring during the induction phase. As mirikizumab’s clinical use expands following the CD indication approval, ongoing pharmacovigilance will be essential to characterise its long-term safety profile.

## 5. Summary

This first multi-database pharmacovigilance study of mirikizumab did not confirm the FDA-flagged hepatotoxicity signal. Potential signals warranting further investigation include thromboembolic events and pulmonary toxicity.

**SIGNIFICANCE OF THIS STUDY.**

**What is already known about this subject?**

• Mirikizumab, a first-in-class interleukin-23p19 antagonist, was approved for ulcerative colitis (2023) and Crohn’s disease (2025).
• The US Food and Drug Administration (FDA) identified a hepatotoxicity signal during pre-approval review, mandating post-marketing surveillance. No independent pharmacovigilance analysis has been published.

**What are the new findings?**

• Disproportionality analysis of the FAERS and JADER revealed that hepatotoxicity showed no disproportionate reporting in either FAERS (ROR 1.19, 0.74–1.92; *n* = 17) or JADER (ROR 0.24, 0.05–1.19; *n* = 1).
• Nine signals met all four criteria in FAERS, including spontaneous abortion (ROR 10.16, 95% CI 5.16–20.02), pulmonary embolism (ROR 5.56, 2.93–10.56) and injection site reactions.
• Comparator analysis identified cytomegalovirus infection and interstitial lung disease as mirikizumab-specific versus the IL-23 class. Weibull analysis (β = 0.65) indicated early-onset adverse event clustering.

**How might it impact clinical practice in the foreseeable future?**

• This first multi-database pharmacovigilance study of mirikizumab did not confirm the FDA-flagged hepatotoxicity signal.
• Potential signals warranting further investigation include thromboembolic events and pulmonary toxicity.

## Figures and Tables

**Table 1:** Demographic characteristics of patients with adverse event reports for IL-23/IL-12/23 inhibitors in FAERS (Q4 2023–Q4 2025).

Characteristic	Mirikizumab (N = 564)	Risankizumab (N = 35,660)	Guselkumab (N = 11,268)	Ustekinumab (N = 10,943)
Female, *n* (%)	242 (42.9)	18,974 (53.2)	4763 (42.3)	4568 (41.7)
Male, *n* (%)	224 (39.7)	14,414 (40.4)	3246 (28.8)	3131 (28.6)
Age, median (IQR)	49 (34–65)	61 (47–70)	53 (41–64)	49 (33–63)
Top reporting country	US (313)	US (28,445)	US (8948)	US (6571)

IQR, interquartile range; US, United States. Sex data missing for some reports.

**Table 2:** Primary disproportionality analysis: signals of disproportionate reporting for mirikizumab in FAERS (all four algorithms concordant).

Preferred Term	*n*	ROR (95% CI)	PRR	χ^2^	IC (IC025)	EBGM (EB05)	Clinical Note
Colitis ulcerative *	78	38.41 (30.24–48.78)	33.06	2419.6	5.05 (4.71)	31.61 (25.97)	Underlying disease
Inj. site hypersensitivity	3	22.38 (7.81–64.15)	19.11	—	— (3.24)	— (2.83)	Known AE
Inj. site discomfort	5	13.21 (5.70–30.63)	11.93	—	— (2.67)	— (3.91)	Known AE
Abortion spontaneous	8	10.16 (5.16–20.02)	9.45	—	— (2.45)	— (4.33)	Novel signal
Frequent bowel movements	8	6.89 (3.50–13.56)	6.41	—	— (1.89)	— (2.96)	IBD-related
Injection site pain	62	6.27 (4.82–8.15)	5.65	241.6	2.52 (2.14)	5.61 (4.49)	Known AE
Pulmonary embolism	9	5.56 (2.93–10.56)	5.20	—	— (1.62)	— (2.50)	Novel signal
Maternal exposure †	18	5.22 (3.28–8.29)	4.95	—	— (1.72)	— (3.14)	Contraindicated
Underdose	9	4.88 (2.57–9.27)	4.57	—	— (1.44)	— (2.11)	Medication error

* Underlying disease indication. † Full PT: Maternal exposure during pregnancy. AE, adverse event; CI, confidence interval; EBGM, empirical Bayesian geometric mean; EB05, lower 90% one-sided CI of EBGM; IC, information component; IC025, lower 95% credible interval of IC; IBD, inflammatory bowel disease; Inj., injection; PRR, proportional reporting ratio; ROR, reporting odds ratio; χ^2^, chi-squared. — indicates value not reported for brevity. For each preferred term, the point estimate is shown with its EBGM (EB05) and IC (IC025) lower bounds in parentheses; the complete χ^2^, IC and EBGM values for all reported terms are provided in Supplementary Table S1.

**Table 3:** Active comparator analysis: mirikizumab versus IL-23/IL-12/23 inhibitor class (all four algorithms concordant).

Preferred Term	*n*	ROR (95% CI)	IC025	EB05	Clinical Note
Pulmonary toxicity	3	240.48 (35.45–1631.44)	4.28	6.46	Mirikizumab-specific
Accidental underdose	3	144.29 (28.41–732.87)	4.11	5.99	Medication error
Inj. site hypersensitivity	3	65.58 (17.13–251.15)	3.69	4.87	Higher than class
CMV infection	4	26.54 (9.39–75.04)	3.09	4.77	Noteworthy
Inj. site discomfort	5	24.20 (9.53–61.46)	3.10	5.63	Higher than class
Colitis ulcerative	78	22.42 (17.34–29.00)	3.69	13.28	IBD indication
Injection site pain	62	13.76 (10.43–18.14)	3.09	8.74	Higher than class
Interstitial lung disease	5	12.49 (5.14–30.38)	2.35	3.43	Mirikizumab-specific
Flushing	8	9.24 (4.56–18.73)	2.16	3.73	Infusion-related
Pulmonary embolism	9	5.96 (3.08–11.53)	1.63	2.59	Confirmed vs. class
Maternal exposure *	18	5.62 (3.49–9.04)	1.75	3.25	Higher than class
Incorrect dose administered	16	3.90 (2.37–6.43)	1.22	2.14	Medication error

* Full PT: Maternal exposure during pregnancy. CMV, cytomegalovirus; CI, confidence interval; EB05, lower 90% one-sided CI of EBGM; IC025, lower 95% credible interval of IC; Inj., injection; ROR, reporting odds ratio. Comparator class = risankizumab + guselkumab + ustekinumab. Where a point estimate is shown as “—”, the corresponding EB05 or IC025 lower bound is given in parentheses; the full χ^2^, IC and EBGM values for all comparator preferred terms are provided in Supplementary Table S3.

**Table 4:** Hepatotoxicity composite analysis: multi-database comparison of IL-23/IL-12/23 inhibitors.

Drug	FAERS *n*	FAERS ROR (95% CI)	FAERS Signal	JADER *n*	JADER ROR (95% CI)	JADER Signal
Mirikizumab	17	1.19 (0.74–1.92)	No	1	0.24 (0.05–1.19)	No
Risankizumab	535	0.57 (0.52–0.62)	No	1	0.16 (0.03–0.78)	No
Guselkumab	359	1.23 (1.11–1.37)	No	12	1.12 (0.63–1.98)	No
Ustekinumab	101	0.35 (0.29–0.42)	No	31	0.67 (0.47–0.95)	No

Signal defined as concordance of all four algorithms (ROR, PRR, BCPNN, EBGM). Hepatotoxicity composite includes SMQ preferred terms for hepatobiliary disorders. CI, confidence interval; ROR, reporting odds ratio; PRR, proportional reporting ratio; BCPNN, Bayesian confidence propagation neural network; EBGM, empirical Bayesian geometric mean; FAERS, FDA Adverse Event Reporting System; JADER, Japanese Adverse Drug Event Report database.

## Data Availability

Analysis code is available upon reasonable request from the corresponding author.

## Supplementary Materials

The following supporting information can be downloaded at: https://www.mdpi.com/article/10.3390/bioengineering13070789/s1, Figure S1: Quarterly reporting trend for mirikizumab in FAERS (Q4 2023–Q4 2025); Table S1: Complete list of mirikizumab signals meeting the reporting odds ratio (ROR) criterion in the primary disproportionality analysis (FAERS); Table S2: Complete list of mirikizumab signals meeting the ROR criterion in the active comparator reference disproportionality analysis (vs. IL-23/IL-12/23 inhibitor class); Table S3: Hepatotoxicity composite analysis: individual preferred term results across IL-23/IL-12/23 inhibitors in FAERS; Table S4: Case-level details of the 17 mirikizumab hepatotoxicity reports identified in FAERS; Table S5: Complete list of mirikizumab signals meeting the ROR criterion in JADER; Table S6: Sensitivity analysis: indication-restricted disproportionality analysis (mirikizumab reports with ulcerative colitis as the recorded indication); Table S7: Sensitivity analysis: age-stratified disproportionality analysis for prespecified preferred terms; Table S8: Sensitivity analysis: sex-stratified disproportionality analysis for prespecified preferred terms.
