# Supplementary material for: Post-Marketing Safety Profile of Mirikizumab: A Multi-Database Pharmacovigilance Study Using FAERS and JADER with IL-23 Inhibitor Class Comparison
Source: Bioengineering (Basel). 2026 Jul 9;13(7):789. doi: 10.3390/bioengineering13070789 (PMC13405810; doi:10.3390/bioengineering13070789)
Supplement: Supplementary file 1 [file bioengineering-13-00789-s001.zip › bioengineering-4383463-supplementary.pdf]

Table S1. Complete list of mirikizumab signals meeting the reporting odds ratio (ROR) criterion in the primary disproportionality analysis (FAERS).

Preferred Term	n	ROR	95% CI	PRR	χ²	IC025	EB05	Sig.ROR	Sig.PRR	Sig.IC	Sig.EBGM	All 4
**Colitis Ulcerative**	**78**	**38.41**	**30.24-48.78**	**33.06**	**2419.6**	**4.71**	**25.966**	**1**	**1**	**1**	**1**	**1**
**Injection Site Hypersensitivity**	**3**	**22.38**	**7.81-64.15**	**19.11**	**51.3**	**3.235**	**2.834**	**1**	**1**	**1**	**1**	**1**
**Injection Site Discomfort**	**5**	**13.21**	**5.70-30.63**	**11.93**	**50**	**2.667**	**3.913**	**1**	**1**	**1**	**1**	**1**
Pulmonary Toxicity	3	11.77	4.11-33.71	10.05	24.4	2.314	1.162	1	1	1	0	0
**Abortion Spontaneous**	**8**	**10.16**	**5.16-20.02**	**9.45**	**60.5**	**2.452**	**4.333**	**1**	**1**	**1**	**1**	**1**
Colon Cancer	3	9.62	3.36-27.54	8.22	19	2.024	0.943	1	1	1	0	0
Ileus	3	9.34	3.26-26.75	7.98	18.3	1.983	0.918	1	1	1	0	0
Cytomegalovirus Infection	4	9.14	3.61-23.13	8.08	24.8	2.057	1.695	1	1	1	0	0
**Frequent Bowel Movements**	**8**	**6.89**	**3.50-13.56**	**6.41**	**36.6**	**1.892**	**2.955**	**1**	**1**	**1**	**1**	**1**
Cerebral Infarction	3	6.73	2.35-19.25	5.75	11.8	1.51	0.699	1	1	1	0	0
**Injection Site Pain**	**62**	**6.27**	**4.82-8.15**	**5.65**	**241.6**	**2.139**	**4.494**	**1**	**1**	**1**	**1**	**1**
Deep Vein Thrombosis	5	5.77	2.49-13.38	5.22	17.1	1.478	1.309	1	1	1	0	0
**Pulmonary Embolism**	**9**	**5.56**	**2.93-10.56**	**5.2**	**30.6**	**1.622**	**2.496**	**1**	**1**	**1**	**1**	**1**
**Maternal Exposure During Pregnancy**	**18**	**5.22**	**3.28-8.29**	**4.95**	**57**	**1.719**	**3.137**	**1**	**1**	**1**	**1**	**1**
Flushing	8	4.94	2.51-9.72	4.6	22.6	1.414	1.926	1	1	1	0	0
**Underdose**	**9**	**4.88**	**2.57-9.27**	**4.57**	**25.1**	**1.435**	**2.105**	**1**	**1**	**1**	**1**	**1**
Haematochezia	7	3.89	1.89-8.00	3.6	13.2	1.028	1.201	1	1	1	0	0
Feeling Hot	5	3.85	1.66-8.91	3.48	8.8	0.894	0.834	1	1	1	0	0
Pancreatitis	4	3.76	1.49-9.51	3.33	6.5	0.78	0.649	1	1	1	0	0
Arrhythmia	3	3.68	1.29-10.53	3.15	4.4	0.643	0.481	1	1	1	0	0
Interstitial Lung Disease	5	3.68	1.59-8.52	3.33	8.1	0.829	0.802	1	1	1	0	0
C-Reactive Protein Increased	4	3.54	1.40-8.95	3.13	5.8	0.693	0.623	1	1	1	0	0
Therapeutic Response Decreased	6	3.52	1.62-7.62	3.23	9.2	0.83	0.923	1	1	1	0	0
Injection Site Bruising	7	3.11	1.51-6.39	2.88	8.6	0.706	0.941	1	1	1	0	0
Upper Respiratory Tract Infection	4	3.09	1.22-7.81	2.73	4.4	0.496	0.57	1	1	1	0	0
Injection Site Haemorrhage	7	3	1.46-6.16	2.78	8	0.653	0.909	1	1	1	0	0
Aspartate Aminotransferase Increased	3	2.99	1.04-8.54	2.55	2.8	0.342	0.434	1	0	1	0	0

Rash	20	1.58	1.02-2.46	1.53	3.7	0.046	0.904	1	0	1	0	0

All preferred terms (PTs) with ROR lower 95% CI > 1 and case count ≥ 3. Signals meeting all four algorithm criteria are highlighted in bold. CI, confidence interval; EB05, lower 90% one-sided CI of empirical Bayesian geometric mean; IC025, lower 95% credible interval of information component; PRR, proportional reporting ratio; PT, preferred term; ROR, reporting odds ratio. Sig. = signal threshold met (1 = yes, 0 = no).

Table S2. Complete list of mirikizumab signals meeting the ROR criterion in the active comparator reference disproportionality analysis (vs IL-23/IL-12/23 inhibitor class).

Preferred Term	n	ROR	95% CI	IC025	EB05	All 4
**Pulmonary Toxicity**	**3**	**240.48**	**35.45-1631.44**	**4.276**	**6.455**	**1**
**Accidental Underdose**	**3**	**144.29**	**28.41-732.87**	**4.106**	**5.992**	**1**
**Injection Site Hypersensitivity**	**3**	**65.58**	**17.12-251.15**	**3.691**	**4.874**	**1**
**Cytomegalovirus Infection**	**4**	**26.54**	**9.39-75.04**	**3.088**	**4.766**	**1**
**Injection Site Discomfort**	**5**	**24.2**	**9.53-61.46**	**3.098**	**5.626**	**1**
**Colitis Ulcerative**	**78**	**22.42**	**17.34-29.00**	**3.686**	**13.276**	**1**
**Injection Site Pain**	**62**	**13.76**	**10.43-18.14**	**3.089**	**8.743**	**1**
Cerebral Infarction	3	13.61	4.45-41.61	2.229	1.256	0
**Interstitial Lung Disease**	**5**	**12.49**	**5.14-30.38**	**2.35**	**3.426**	**1**
Ileus	3	10.15	3.38-30.55	1.881	0.94	0
**Flushing**	**8**	**9.24**	**4.56-18.73**	**2.157**	**3.731**	**1**
Therapy Non-Responder	3	9.13	3.05-27.31	1.749	0.858	0
Arrhythmia	3	8.9	2.98-26.61	1.718	0.841	0
Aspartate Aminotransferase Increased	3	7.28	2.46-21.60	1.465	0.721	0
Blood Pressure Decreased	3	6.38	2.16-18.84	1.295	0.657	0
C-Reactive Protein Increased	4	5.99	2.31-15.54	1.33	0.935	0
**Pulmonary Embolism**	**9**	**5.96**	**3.08-11.53**	**1.626**	**2.589**	**1**
Deep Vein Thrombosis	5	5.65	2.39-13.38	1.341	1.217	0
**Maternal Exposure During Pregnancy**	**18**	**5.62**	**3.49-9.04**	**1.747**	**3.251**	**1**
Feeling Hot	5	5.38	2.27-12.74	1.277	1.148	0
Injection Site Bruising	7	5.3	2.53-11.09	1.382	1.763	0
Abortion Spontaneous	8	5.05	2.52-10.10	1.364	1.901	0
Paraesthesia	6	4.01	1.82-8.82	0.95	1.032	0
**Incorrect Dose Administered**	**16**	**3.9**	**2.37-6.43**	**1.224**	**2.14**	**1**
Therapeutic Response Decreased	6	3.78	1.72-8.31	0.871	0.972	0
Cardiac Failure	4	3.5	1.36-8.98	0.611	0.611	0

Discomfort	4	3.4	1.32-8.72	0.57	0.6	0
Pancreatitis	4	3.32	1.29-8.53	0.54	0.592	0
Drug Hypersensitivity	4	3.1	1.21-7.95	0.446	0.567	0
Upper Respiratory Tract Infection	4	3.06	1.19-7.84	0.427	0.563	0
Injection Site Swelling	5	3.06	1.30-7.17	0.512	0.685	0
Myalgia	6	3	1.37-6.58	0.554	0.79	0
Rash Pruritic	3	2.96	1.02-8.60	0.272	0.43	0
Asthma	3	2.94	1.01-8.53	0.261	0.429	0
Frequent Bowel Movements	8	2.79	1.41-5.55	0.557	0.933	0
Chest Discomfort	5	2.79	1.19-6.52	0.384	0.642	0
Hypersensitivity	5	2.76	1.18-6.46	0.371	0.638	0
Underdose	9	2.68	1.40-5.14	0.54	0.977	0
Rash	20	2.65	1.69-4.14	0.744	1.481	0
Headache	22	2.63	1.71-4.03	0.755	1.521	0
Alopecia	6	2.47	1.13-5.40	0.285	0.682	0
Haematochezia	7	2.19	1.06-4.53	0.173	0.698	0
Pyrexia	9	2.03	1.06-3.87	0.15	0.77	0
Dizziness	12	1.91	1.09-3.37	0.16	0.858	0
Drug Ineffective	35	1.6	1.14-2.25	0.162	1.058	0

Active comparator was the combined cohort of risankizumab, guselkumab, and ustekinumab. Signals meeting all four algorithms in bold. All 4 = signal met by all four algorithms (ROR, PRR, BCPNN, EBGM); 1 = yes, 0 = no.

Table S3. Hepatotoxicity composite analysis: individual preferred term results across IL-23/IL-12/23 inhibitors in FAERS.

Drug	Preferred Term	n	ROR	95% CI	IC025	EB05	All 4
Mirikizumab	Hepato_Composite	17	1.194	0.74–1.92	-0.391	0.676	0
Mirikizumab	Drug-Induced Liver Injury	2	1.496	0.43–5.18	-0.798	0.242	0
Mirikizumab	Hepatotoxicity	1	1.945	0.39–9.66	-0.634	0.16	0
Mirikizumab	Hepatic Failure	1	2.057	0.41–10.22	-0.553	0.163	0
Mirikizumab	Alanine Aminotransferase Increased	2	1.494	0.43–5.18	-0.8	0.242	0
Mirikizumab	Aspartate Aminotransferase Increased	3	2.414	0.84–6.91	0.036	0.394	0
Mirikizumab	Hepatic Enzyme Increased	3	1.196	0.42–3.42	-0.972	0.287	0
Mirikizumab	Liver Function Test Increased	1	1.266	0.26–6.29	-1.252	0.142	0
Mirikizumab	Blood Bilirubin Increased	1	2.181	0.44–10.84	-0.469	0.165	0
Mirikizumab	Cholestasis	2	4.215	1.22–14.61	0.692	0.346	0
Mirikizumab	Autoimmune Hepatitis	1	6.034	1.21–29.99	0.995	0.204	0
Mirikizumab	Liver Disorder	2	1.477	0.43–5.12	-0.817	0.241	0
Mirikizumab	Hepatic Steatosis	1	2.212	0.45–10.99	-0.448	0.166	0
Mirikizumab	Hepatomegaly	1	6.637	1.33–32.99	1.132	0.207	0
Mirikizumab	Gamma-Glutamyltransferase Increased	1	2.906	0.58–14.44	-0.056	0.176	0

Mirikizumab	Blood Alkaline Phosphatase Increased	1	2.653	0.53–13.18	-0.187	0.173	0
Risankizumab	Hepato_Composite	535	0.566	0.52–0.62	-0.918	0.535	0
Risankizumab	Drug-Induced Liver Injury	23	0.22	0.15–0.33	-2.717	0.157	0
Risankizumab	Hepatotoxicity	11	0.233	0.13–0.42	-2.84	0.143	0
Risankizumab	Hepatitis	7	0.143	0.07–0.29	-3.698	0.079	0
Risankizumab	Hepatic Failure	33	0.724	0.52–1.02	-0.927	0.518	0
Risankizumab	Alanine Aminotransferase Increased	42	0.398	0.29–0.54	-1.732	0.307	0
Risankizumab	Aspartate Aminotransferase Increased	25	0.275	0.19–0.41	-2.377	0.198	0
Risankizumab	Transaminases Increased	9	0.243	0.13–0.46	-2.848	0.141	0
Risankizumab	Hepatic Enzyme Increased	134	0.724	0.61–0.86	-0.698	0.622	0
Risankizumab	Liver Function Test Abnormal	21	0.758	0.50–1.16	-0.968	0.489	0
Risankizumab	Liver Function Test Increased	59	0.793	0.61–1.02	-0.686	0.62	0
Risankizumab	Blood Bilirubin Increased	21	0.491	0.32–0.75	-1.588	0.331	0
Risankizumab	Hyperbilirubinaemia	3	0.162	0.06–0.46	-3.831	0.066	0
Risankizumab	Jaundice	24	0.693	0.47–1.03	-1.063	0.466	0
Risankizumab	Cholestasis	5	0.145	0.06–0.33	-3.803	0.072	0
Risankizumab	Cholestatic Liver Injury	1	0.226	0.05–1.12	-3.723	0.048	0
Risankizumab	Autoimmune Hepatitis	12	0.792	0.45–1.38	-1.065	0.431	0
Risankizumab	Liver Disorder	117	1.1	0.92–1.32	-0.122	0.919	0
Risankizumab	Liver Injury	26	0.273	0.19–0.40	-2.378	0.198	0
Risankizumab	Hepatic Steatosis	66	1.563	1.23–1.99	0.295	1.197	0
Risankizumab	Hepatomegaly	21	1.512	0.99–2.32	0.01	0.887	0
Risankizumab	Gamma-Glutamyltransferase Increased	8	0.258	0.13–0.51	-2.804	0.144	0
Risankizumab	Blood Alkaline Phosphatase Increased	17	0.486	0.30–0.78	-1.66	0.312	0
Guselkumab	Hepato_Composite	359	1.232	1.11–1.37	0.141	1.111	0
Guselkumab	Drug-Induced Liver Injury	2	0.074	0.02–0.26	-5.11	0.02	0
Guselkumab	Hepatotoxicity	2	0.162	0.05–0.56	-3.994	0.053	0
Guselkumab	Hepatitis	9	0.578	0.31–1.09	-1.609	0.302	0
Guselkumab	Hepatic Failure	11	0.788	0.44–1.41	-1.098	0.416	0
Guselkumab	Alanine Aminotransferase Increased	33	1.002	0.71–1.41	-0.462	0.696	0
Guselkumab	Aspartate Aminotransferase Increased	19	0.67	0.43–1.05	-1.172	0.427	0
Guselkumab	Transaminases Increased	16	1.352	0.83–2.19	-0.213	0.736	0
Guselkumab	Hepatic Enzyme Increased	129	2.242	1.88–2.67	0.903	1.876	0
Guselkumab	Liver Function Test Abnormal	19	2.195	1.41–3.43	0.527	1.219	0
Guselkumab	Liver Function Test Increased	32	1.377	0.97–1.95	-0.014	0.922	0

Guselkumab	Blood Bilirubin Increased	9	0.69	0.36–1.31	-1.355	0.348	0
Guselkumab	Jaundice	1	0.134	0.03–0.67	-4.477	0.024	0
Guselkumab	Cholestasis	2	0.21	0.06–0.72	-3.619	0.07	0
Guselkumab	Autoimmune Hepatitis	7	1.511	0.74–3.10	-0.319	0.545	0
Guselkumab	Liver Disorder	55	1.649	1.26–2.15	0.347	1.226	0
Guselkumab	Liver Injury	7	0.246	0.12–0.50	-2.926	0.132	0
Guselkumab	Hepatic Steatosis	31	2.341	1.65–3.33	0.737	1.547	0
Guselkumab	Hepatomegaly	6	1.44	0.67–3.11	-0.444	0.484	0
Guselkumab	Gamma-Glutamyltransferase Increased	4	0.435	0.17–1.10	-2.312	0.177	0
Guselkumab	Blood Alkaline Phosphatase Increased	4	0.397	0.16–1.00	-2.443	0.165	0
Ustekinumab	Hepato_Composite	101	0.349	0.29–0.42	-1.766	0.3	0
Ustekinumab	Drug-Induced Liver Injury	11	0.353	0.20–0.63	-2.252	0.209	0
Ustekinumab	Hepatotoxicity	3	0.233	0.08–0.67	-3.318	0.093	0
Ustekinumab	Hepatitis	2	0.156	0.04–0.54	-4.041	0.052	0
Ustekinumab	Alanine Aminotransferase Increased	7	0.23	0.11–0.47	-3.022	0.124	0
Ustekinumab	Aspartate Aminotransferase Increased	5	0.194	0.08–0.45	-3.388	0.095	0
Ustekinumab	Hepatic Enzyme Increased	30	0.535	0.38–0.76	-1.381	0.384	0
Ustekinumab	Liver Function Test Abnormal	6	0.748	0.35–1.62	-1.381	0.314	0
Ustekinumab	Liver Function Test Increased	12	0.543	0.31–0.95	-1.607	0.313	0
Ustekinumab	Cholestasis	4	0.389	0.15–0.98	-2.472	0.162	0
Ustekinumab	Autoimmune Hepatitis	3	0.724	0.25–2.07	-1.69	0.219	0
Ustekinumab	Liver Disorder	15	0.47	0.29–0.77	-1.746	0.293	0
Ustekinumab	Liver Injury	6	0.219	0.10–0.47	-3.146	0.113	0
Ustekinumab	Hepatic Steatosis	11	0.874	0.49–1.56	-0.951	0.451	0
Ustekinumab	Hepatomegaly	1	0.341	0.07–1.69	-3.138	0.071	0
Ustekinumab	Gamma-Glutamyltransferase Increased	1	0.149	0.03–0.74	-4.326	0.028	0

Individual hepatic preferred terms comprising the composite hepatotoxicity endpoint, by drug. No individual hepatic preferred term met the all-four-algorithm signal criterion for mirikizumab. HEPATO_COMPOSITE = composite endpoint comprising all listed PTs combined.

Table S4. Case-level details of the 17 mirikizumab hepatotoxicity reports identified in FAERS.

**Case ID**	**Sex**	**Age**	**Country**	**Event date**	**Hepatic PTs**	**Indication**	**Concomitant medications**
259805121			US	20250924	HEPATIC FAILURE	Product used for unknown indication	
243124611	M	38	DE	20240601	AUTOIMMUNE HEPATITIS	Colitis ulcerative	
239059111	F	65	JP		LIVER DISORDER	Colitis ulcerative	LIALDA

239124713	F	79	DE	20240511	CHOLESTASIS	Colitis ulcerative; Product used for unknown indication	BUDESONIDE; CALCIUM; LOPERAMIDE; MESALAMINE; METOPROLOL; MIRTAZAPINE; URSODIOL
251384841	F	57	US	20240501	ALANINE AMINOTRANSFERASE INCREASED; ASPARTATE AMINOTRANSFERASE INCREASED	Product used for unknown indication	
236189483	M	75	JP	20240306	LIVER DISORDER	Colitis ulcerative	IMURAN; LIALDA; BUDESONIDE
256753801	F	45	US		BLOOD BILIRUBIN INCREASED	Product used for unknown indication	OMVOH
253895021	M	48	US	20250521	HEPATOMEGALY	Crohn's disease	
257997611	F		US		HEPATIC ENZYME INCREASED	Colitis ulcerative	
259470091			US	20250922	HEPATIC ENZYME INCREASED	Colitis ulcerative	
242449392	M	55	CH	20240507	ALANINE AMINOTRANSFERASE INCREASED; BLOOD ALKALINE PHOSPHATASE INCREASED; GAMMA-GLUTAMYLTRANSFERASE INCREASED; ASPARTATE AMINOTRANSFERASE INCREASED	Colitis ulcerative; Product used for unknown indication	PREDNISOLONE; BUDESONIDE
259851792	F	53	US		DRUG-INDUCED LIVER INJURY	Colitis ulcerative	
254112791			US		LIVER FUNCTION TEST INCREASED	Product used for unknown indication	
261418841			US	20251128	HEPATIC ENZYME INCREASED; HEPATOTOXICITY	Inflammatory bowel disease	
241994041	M	75	JP	20240109	DRUG-INDUCED LIVER INJURY	Colitis ulcerative; Product used for unknown indication	CALONAL; LIALDA; PREDNISOLONE
251370011	F	46	US	20241004	HEPATIC STEATOSIS; ASPARTATE AMINOTRANSFERASE INCREASED	Colitis ulcerative; Pain management; Anaemia; Insomnia; Product used for unknown indication	OXYCODONE; FERROUS SULFATE; MELATONIN; ASCORBIC ACID; BUDESONIDE\FORMOTEROL; PREDNISONE; NALTREXONE; NEURONTIN; BUPROPION; COMBIVENT RESPIMAT
242449311	F		AT		CHOLESTASIS	Product used for unknown indication	MIRIKIZUMAB

Individual case demographics, hepatic preferred terms, indication, and concomitant medications. AT, Austria; CH,

Switzerland; DE, Germany; JP, Japan; PT, preferred term; US, United States. Empty cells indicate missing data in the original FAERS report.

Table S5. Complete list of mirikizumab signals meeting the ROR criterion in JADER.

Preferred Term (J-MedDRA)	n	ROR	95% CI	IC025	EB05	All 4
**潰瘍性大腸炎**	**31**	**250.29**	**166.47-376.32**	**6.959**	**83.498**	**1**
**薬物不耐性**	**4**	**135.07**	**52.32-348.71**	**5.837**	**10.999**	**1**
心不全	4	3.75	1.46-9.62	0.718	0.642	0
肺炎	7	3.12	1.49-6.54	0.633	0.922	0
アナフィラキシーショック	5	2.48	1.05-5.82	0.201	0.593	0

JADER analysis period covered the same time window as FAERS. Preferred terms in original Japanese MedDRA coding. JADER, Japanese Adverse Drug Event Report database; J-MedDRA, Japanese Medical Dictionary for Regulatory Activities. Preferred term translations: 潰瘍性大腸炎 = ulcerative colitis; 薬物不耐性 = drug intolerance; 肺炎 = pneumonia; 間質性肺疾患 = interstitial lung disease; アナフィラキシーショック = anaphylactic shock.

Table S6. Sensitivity analysis: indication-restricted disproportionality analysis (mirikizumab reports with ulcerative colitis as the recorded indication).

Preferred Term	n	ROR	95% CI	All 4
**Colitis Ulcerative**	**70**	**43.71**	**33.67-56.75**	**1**
**Pulmonary Toxicity**	**3**	**14.72**	**4.72-45.89**	**1**
**Cytomegalovirus Infection**	**4**	**12.24**	**4.57-32.82**	**1**
**Colon Cancer**	**3**	**12.21**	**3.92-38.07**	**1**
**Abortion Spontaneous**	**6**	**10.84**	**4.84-24.30**	**1**
**Deep Vein Thrombosis**	**5**	**7.9**	**3.27-19.10**	**1**
**Pulmonary Embolism**	**8**	**6.93**	**3.44-13.98**	**1**
**Injection Site Pain**	**41**	**6.15**	**4.45-8.52**	**1**
**Underdose**	**8**	**6.01**	**2.98-12.12**	**1**
**Frequent Bowel Movements**	**6**	**5.57**	**2.49-12.49**	**1**
**Maternal Exposure During Pregnancy**	**13**	**5.39**	**3.10-9.38**	**1**
**Flushing**	**5**	**4.31**	**1.78-10.43**	**1**
Accidental Underdose	3	4.16	1.33-12.95	0
C-Reactive Protein Increased	4	4.12	1.54-11.05	0
Interstitial Lung Disease	4	3.96	1.48-10.60	0
Therapeutic Response Decreased	5	3.93	1.63-9.50	0
Injection Site Haemorrhage	6	3.72	1.66-8.34	0
Injection Site Bruising	6	3.71	1.66-8.31	0
Upper Respiratory Tract Infection	4	3.24	1.21-8.67	0
Haematochezia	5	3.17	1.31-7.67	0
Incorrect Dose Administered	12	1.81	1.02-3.21	0

All preferred terms with ROR lower 95% CI > 1 and case count ≥ 3 within the UC-restricted cohort. All 4 = signal met by all four algorithms (1 = yes, 0 = no). The UC-restricted analysis reproduced all nine signals from the primary analysis and identified three additional signals (pulmonary toxicity, cytomegalovirus infection, deep vein thrombosis), strengthening the thromboembolic and pulmonary signals.

Table S7. Sensitivity analysis: age-stratified disproportionality analysis for prespecified preferred terms.

Age group	Preferred Term	n	ROR	95% CI	All 4
**<=64**	**Colitis Ulcerative**	**41**	**19.89**	**14.28-27.70**	**1**
**<=64**	**Injection Site Pain**	**38**	**4.96**	**3.52-6.98**	**1**
<=64	Pulmonary Embolism	3	3.05	0.98-9.52	0
**<=64**	**Abortion Spontaneous**	**6**	**9.36**	**4.17-21.03**	**1**
**<=64**	**Maternal Exposure During Pregnancy**	**12**	**4.09**	**2.29-7.29**	**1**
<=64	Frequent Bowel Movements	4	3.45	1.29-9.26	0
**<=64**	**Underdose**	**7**	**6.48**	**3.06-13.74**	**1**
<=64	Cytomegalovirus Infection	2	5.12	1.27-20.60	0
**>64**	**Colitis Ulcerative**	**26**	**98.74**	**63.48-153.57**	**1**
>64	Injection Site Pain	2	1.03	0.25-4.16	0
**>64**	**Pulmonary Embolism**	**4**	**7.97**	**2.94-21.64**	**1**
**>64**	**Frequent Bowel Movements**	**3**	**10.26**	**3.26-32.35**	**1**
>64	Underdose	1	3.76	0.52-26.95	0
>64	Cytomegalovirus Infection	2	23.01	5.67-93.39	0
>64	Interstitial Lung Disease	1	1.59	0.22-11.42	0

Subgroups defined as ≤64 years and >64 years based on FAERS-recorded patient age. All 4 = signal met by all four algorithms (1 = yes, 0 = no). The pulmonary embolism signal was driven predominantly by older patients (>64 years), whereas the injection site pain signal was concentrated in younger patients (≤64 years).

Table S8. Sensitivity analysis: sex-stratified disproportionality analysis for prespecified preferred terms.

Sex	Preferred Term	n	ROR	95% CI	All 4
**F**	**Colitis Ulcerative**	**31**	**24.98**	**17.12-36.43**	**1**
**F**	**Injection Site Pain**	**40**	**7.6**	**5.42-10.67**	**1**
**F**	**Pulmonary Embolism**	**4**	**5.61**	**2.09-15.08**	**1**
**F**	**Abortion Spontaneous**	**8**	**12.19**	**6.02-24.68**	**1**
**F**	**Maternal Exposure During Pregnancy**	**18**	**6.71**	**4.15-10.86**	**1**
**F**	**Underdose**	**6**	**5.85**	**2.60-13.15**	**1**
F	Cytomegalovirus Infection	1	6.24	0.87-44.53	0
**M**	**Colitis Ulcerative**	**44**	**37.81**	**27.17-52.62**	**1**
**M**	**Injection Site Pain**	**13**	**3.3**	**1.88-5.78**	**1**
**M**	**Pulmonary Embolism**	**4**	**4.66**	**1.73-12.54**	**1**
**M**	**Frequent Bowel Movements**	**7**	**10.09**	**4.75-21.44**	**1**
**M**	**Underdose**	**3**	**6.21**	**1.99-19.42**	**1**
**M**	**Cytomegalovirus Infection**	**3**	**11.44**	**3.66-35.80**	**1**
M	Interstitial Lung Disease	3	3.89	1.24-12.15	0

Female-specific events (spontaneous abortion, maternal exposure during pregnancy) were not assessed in the male subgroup. F, female; M, male. All 4 = signal met by all four algorithms (1 = yes, 0 = no). The pulmonary embolism signal was robust in both sexes (all-four-concordant). The cytomegalovirus infection signal reached all-four-concordance in males only.

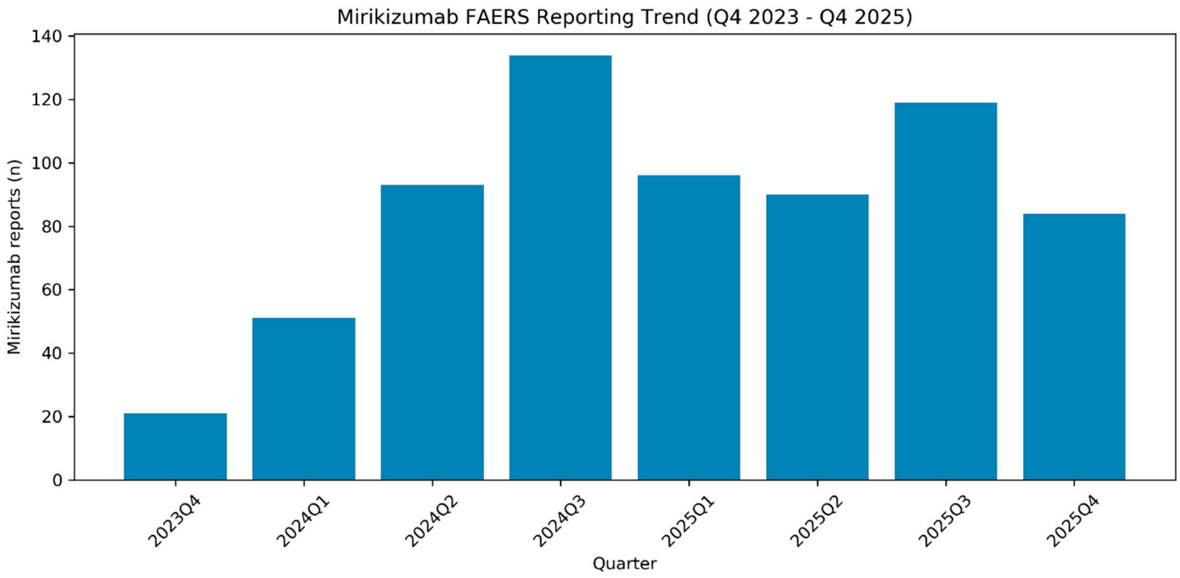

Figure S1. Quarterly reporting trend for mirikizumab in FAERS (Q4 2023–Q4 2025).

Bar chart showing the number of mirikizumab adverse event reports submitted in each quarter from the date of first US approval (Q4

2023) through Q4 2025. Reporting volume increased from 21 reports in Q4 2023 to a peak of 134 reports in Q3 2024, followed by relative stabilisation at 84–119 reports per quarter through Q4 2025. The delayed peak rather than an immediate post-approval surge suggests that the Weber effect had limited influence on the observed signals. Quarterly counts were derived from drug-record matches in FAERS quarterly files; the apparent total (n=688) exceeds the deduplicated primary-suspect cohort (n=564) because individual cases reported in multiple quarterly files (e.g., initial reports with subsequent follow-up reports) are counted in each quarter of submission. Data for Q4 2024 were not available at the time of analysis and are omitted from the figure; this gap does not affect the overall trend interpretation.
